# Defective thymic output in WAS patients is associated with abnormal actin organization

**DOI:** 10.1038/s41598-017-12345-z

**Published:** 2017-09-20

**Authors:** Wenyan Li, Xiaoyu Sun, Jinzhi Wang, Qin Zhao, Rongxin Dai, Yanping Wang, Lina Zhou, Lisa Westerberg, Yuan Ding, Xiaodong Zhao, Chaohong Liu

**Affiliations:** 1 Chongqing Key Laboratory of Child Infection and Immunity, Department of Pediatric Research Institute, Ministry of Education Key Laboratory of Child Development and Disorder, International Science and Technology Cooperation base of Child development and Critical Disorders, Children’s Hospital of Chongqing Medical University, Chongqing, 400014 China; 2 Department of Microbiology, Tumor and Cell Biology, Karolinska Institutet, Stockholm, 17177 Sweden; 3 Department of Pathogen Biology, School of Basic Medicine, Huazhong University of Science and Technology, Wuhan, 430030 China

## Abstract

Wiskott-Aldrich syndrome protein (WASp) is a key regulator of the actin cytoskeleton. Defective T - cell function is a major cause for immune deficiency in Wiskott-Aldrich syndrome (WAS) patients. T cells originate in the bone marrow and develop in the thymus, and then migrate to peripheral tissues. TCR excision circles (TRECs) present in thymic output cells stably, which is used as a molecular marker for thymic output. We found that CD8^+^ T naïve cells of classic WAS patients were significantly reduced, and TRECs in patients with classic WAS and X-linked thrombocytopenia (XLT) dramatically decreased compared with that of HCs. TRECs were also reduced in WAS (KO) mice. These suggest that defective thymic output partially accounts for T cell lymphopenia in WAS patients. However, the correlation between the defect of thymic output and actin organization still remains elusive. We found that the subcellular location and the levels of of F-actin were altered in T cells from both WAS and XLT patients compared to that of HCs with or without stimulation. Our study shows that WASp plays a critical role in thymic output, which highly correlates with the subcellular location and level of F-actin in T cells.

## Introduction

Wiskott-Aldrich syndrome (WAS, OMIM#301000) is a rare X-linked recessive immune deficiency characterized by eczema, microthrombocytopenia, and immunodeficiency^1,2^. It is usually classified by the clinical severity score ranges from 1–2 for XLT, mild WAS patients, and 3–4 for classic WAS. A score 5 is associated with patients developing autoimmunity or malignancies^3,4^. The clinical manifestations are caused by mutations in *WAS* gene (Xp11.22–23), which encodes the WAS protein (WASp). WASp is predominantly expressed in hematopoietic cells. WASp is an Arp2/3 activator that control actin assembly downstream of Cdc42 and Rac activation. WASp deficiency causes dysfunction of actin polymerization, and podosome formation, which results in abnormal cell migration^5,6^.

Defective T-cell function has been believed to be a major cause for immune deficiency in WAS^7,8^. T cells go through development in the thymus, and then egress to the blood stream. T-cell receptor (TCR) gene rearrangement produces TCR excision circles (TRECs) that do not replicate during mitosis and can be detected in newly formed T cells. Therefore, the presence of TRECs in circulating T cells indicates the recent thymic output cells^9^. T cell lymphopenia in WAS patients accounting for abnormal T cell proliferation and increased rate of apoptosis has been reported in previous research^10,11^. However, thymic output which is dependent on the normal function of cell migration in WAS has not been examined adequately. Moreover, whether the correlation between thymic output and actin alteration in WAS exists still remains elusive.

In this study, we examined the subsets of T cells in peripheral blood, thymic output and subcellular location of F-actin in T cells from four classic WAS patients and four XLT patients. We also tested the thymic output in WAS knockout (KO) mice. Our results suggest that WASp plays a critical role in thymic output that is highly associated with the subcellular location of F-actin in T cells.

## Results

### Clinical characteristics of WAS and XLT patients with Wiskott-Aldrich syndrome

As a representative of classic WAS patients, P1 presented with thrombocytopenia, severe eczema, recurrent respiratory tract infections from 3 days of age. At the age of 6 months, P1 was diagnosed as a classic WAS and sequencing of the WAS gene identified a splice mutation in intron 8 (IVS8 + 1G > A) that causes exon 8 deletion, resulting in a premature stop signal at amino acid 246. P1 had autoimmune hemolytic anemia (AIHA) with a positive Coombs’ test at 10 months. Then P1 received hematopoietic stem cell transplantation treatment (Table 1).

**Table 1:** Clinical characteristics of eight patients with Wiskott-Aldrich syndrome.

Patient ID	Age at sample collection	Age of onset	Clinical score	Protien expression	WAS gene mutation	Codon change or predicted splicing abnormality
P1	6 m	3d	5 A	Absent	IVS8 + 1 G > A	Predicted splicing abnormality
P2	3 m	15d	3	Absent	IVS8 + 1 G > A	Predicted splicing abnormality
P3	1 y	36d	3	Absent	923–924dupGC	Q310fsX445
P4	2 m	2 m	3	Decreased	1040 T > A	L347X
P5	4 y	9 m	2	Decreased	257 G > A	V75M
P6	4 y	5d	2	Decreased	1453 G > A	D485N
P7	10 m	2 m	2	Normal	1453 G > A	D485N
P8	1 y	5 m	2	Normal	1315 C > T	R439W

As a representative of XLT patients, P5 was diagnosed with low platelet count (40 ~ 80 × 10^9^/L) at 9 months, and no other clinical manifestations before 4 years old. Mutation analysis revealed a missense mutation in exon 2 of the WAS gene at c. 257 G > A (V75 M) (Table 1).

### Abnormal WASp expression in peripheral blood lymphocytes of patients

**Figure 1:**
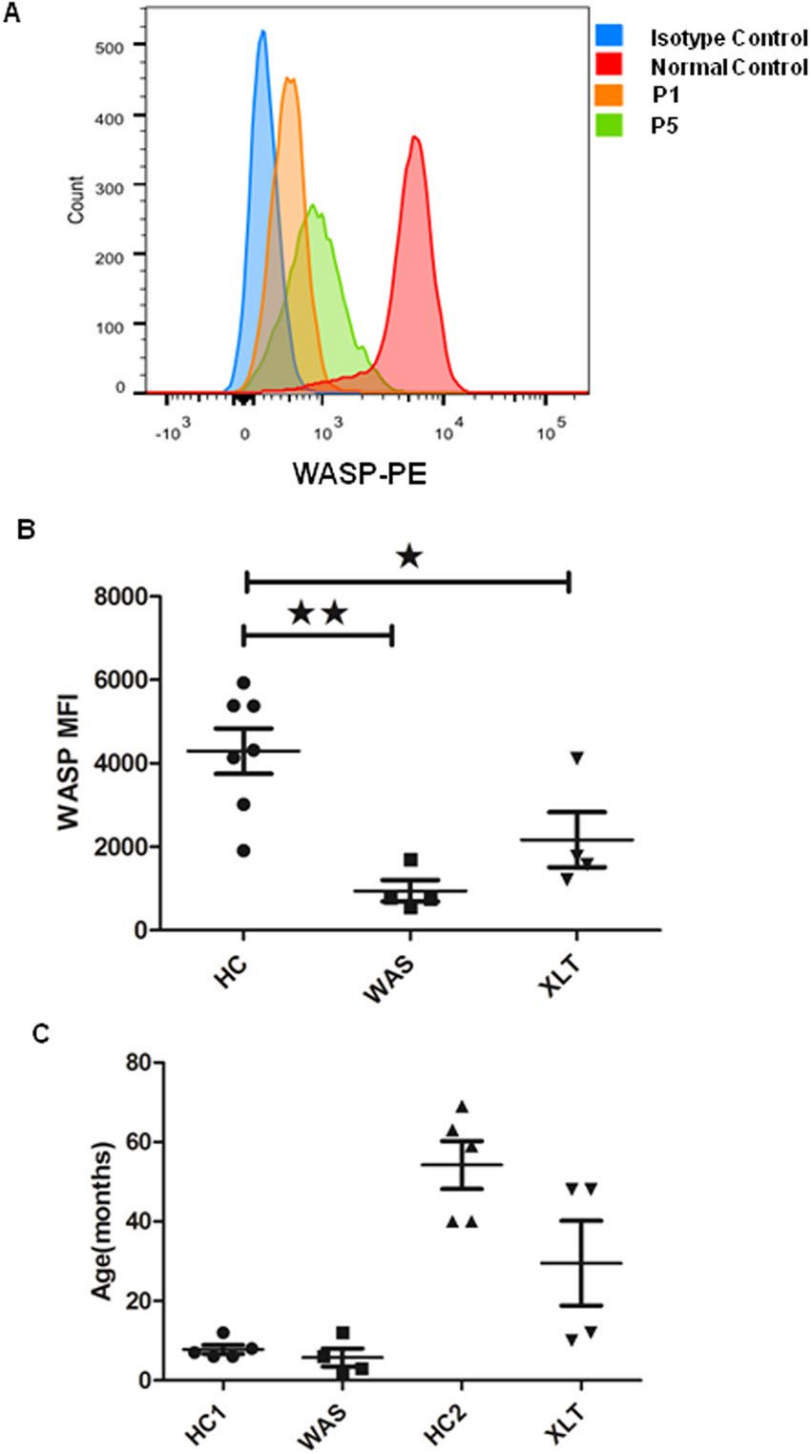
WASp expression in XLT and WAS patients. (**A**) Flow cytometry analysis of the expression of WASp in PBMCs from normal control, WAS patient (P1) and XLT patient (P5). (**B**) The quantification of MFI of WASp from eight patients and healthy controls. (**C**) The age of WAS patients, XLT patients and their respective healthy controls.

To investigate if the *WAS* gene mutations of the patients affect WASp expression, we examined the expression levels of WASp in peripheral blood lymphocytes by flow cytometry. The expression levels of WASp were reduced in XLT patient (P5) and more in WAS patient (P1) when compared with that in the normal control, but higher than that of the isotype control (Fig. 1A). These results can also be seen in other three WAS and three XLT patients and suggest that the expression levels of WASp are inversely related with the severity of WAS (Fig. 1B). In order to minimize the influence of age on the following analysis, we have plotted the ages from healthy controls(HCs) and WAS patients. Based on the age information of XLT and WAS patients, the corresponding HC1 and HC2 are tightly enough matched for comparison (Fig. 1C).

### Decreased percentages and numbers of naïve T cells in peripheral blood of patients

**Figure 2:**
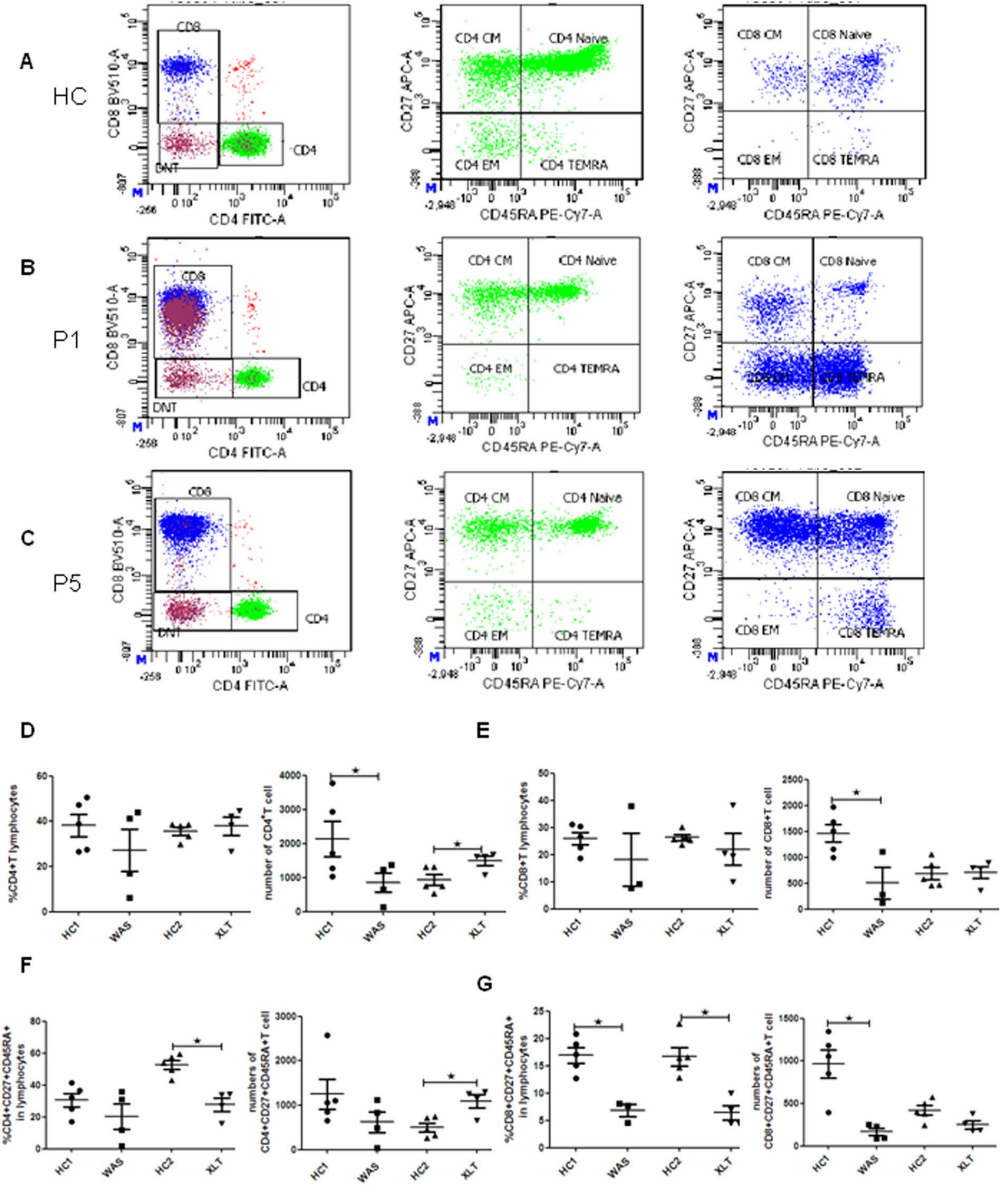
CD4^+^ and CD8^+^ T naïve cells are reduced in XLT and WAS patients. Flow cytometric analysis of CD3^+^ T - cell subsets in peripheral blood. (**A**) Flow cytometric analysis of CD3^+^ T - cell subsets in a healthy control. (**B**) Flow cytometric analysis of CD3^+^ T - cell subsets in P1. (**C**) Flow cytometric analysis of CD3^+^ T - cell subsets in P5. (**D**) Percentage and number of CD4^+^ T cells in WAS patients (P1-4), XLT patients (P5-8) and age-matched HCs (WAS-HC1, XLT-HC2). (**E**) Percentage and number of CD8^+^ T cells in WAS patients (P1-4), XLT patients (P5-8) and age-matched HCs (HC1, HC2). (**F**) Percentage and number of CD4 naïve T cells (CD4^+^CD27^+^CD45RA^+^) in WAS patients (P1-4), XLT patients (P5-8) and age-matched HCs (HC1, HC2). (**G**) Percentage and number of CD8 naïve T cells (CD4^+^CD27^+^CD45RA^+^) in WAS patients (P1-4), XLT patients (P5-8) and age-matched HCs (HC1, HC2).

Many studies have shown that the T - cell defect as a major cause of the immunodeficiency observed in WAS patients^7,8^. To determine which subsets of T cells that are mainly impaired in these patients, we analyzed the percentages and numbers of T - cell subsets in their peripheral blood and healthy controls (HCs) by using flow cytometry (Fig. 2 A-C). We found that WAS patients displayed a profoundly decreased number but normal frequency of CD4^+^ T cells. XLT patients showed a normal frequency but increased number of CD4^+^ T cells within total lymphocytes when compared to HCs (Fig. 2D). The number of CD8^+^ T cells within total lymphocytes was decreased in WAS patients when compared to HCs. (Fig. 2E). Furthermore, we found that XLT patients showed a greatly reduced percentage but increased number in naïve CD4^+^ T cells compared to that of HCs, but no difference was observed in WAS patients (Fig. 2F). The frequency of naïve CD8^+^ T cells was reduced both in WAS and XLT patients, and only the number of CD8^+^ T cells was decreased in WAS patients (Fig. 2G). There were no differences between WAS, XLT and HCs in the percentages or numbers of the central memory T cells (CM), effect memory T cells (EM) and TEMRA T cells in CD4^+^ T cells or CD8^+^ T cells (data not show). These data suggest that WASp deficiency reduces the naïve T cells in peripheral blood, and this reduction is inversely correlated with the level of WASp expression

### Thymic output of T lymphocytes is impaired in WAS and XLT patients

**Figure 3:**
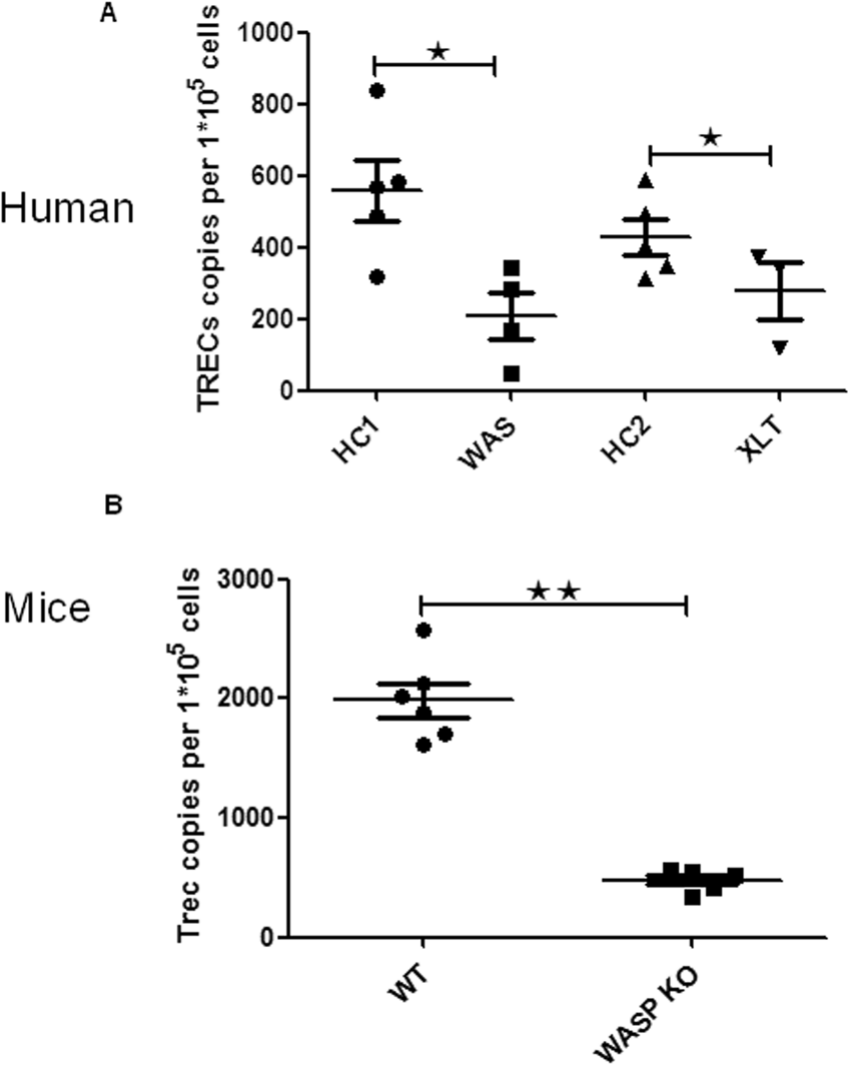
sjTRECs is reduced in WASp deficient T cells. (**A**) Copy numbers of sjTRECs per 1 × 10^5^ CD3^+^ T cells from HCs, WAS (P1-4) and XLT (P5-7) and (**B**) from WT and WAS KO mice.

WASp plays a major role in cell migration by regulating actin polymerization and podosome formation, and we found reduced number of naïve T cells in the WAS patients. Therefore it is highly possible that the thymic output is affected in WAS or XLT patients. To investigate whether WASp deficiency impaired thymic output, we examined the thymic output by using signal joint TRECs (sjTRECs). We ananlyzed the sjTRECs of the patients and found that sjTRECs copies (per 1 × 10^5^ T cells) of WAS and XLT were significantly lower when compared to HCs (Fig. 3A). To further confirm the results, we examined the thymic output in WAS KO mice by measuring the sjTRECs. We found that sjTRECs copies were significantly lower in WAS KO mice when compared to that of WT mice (Fig. 3B). These results indicate that WAS and XLT both have abnormal thymic output due to the WASp deficiency.

### Abnormal expression and subcellular location of F-actin in the T cells of WAS and XLT patients

**Figure 4:**
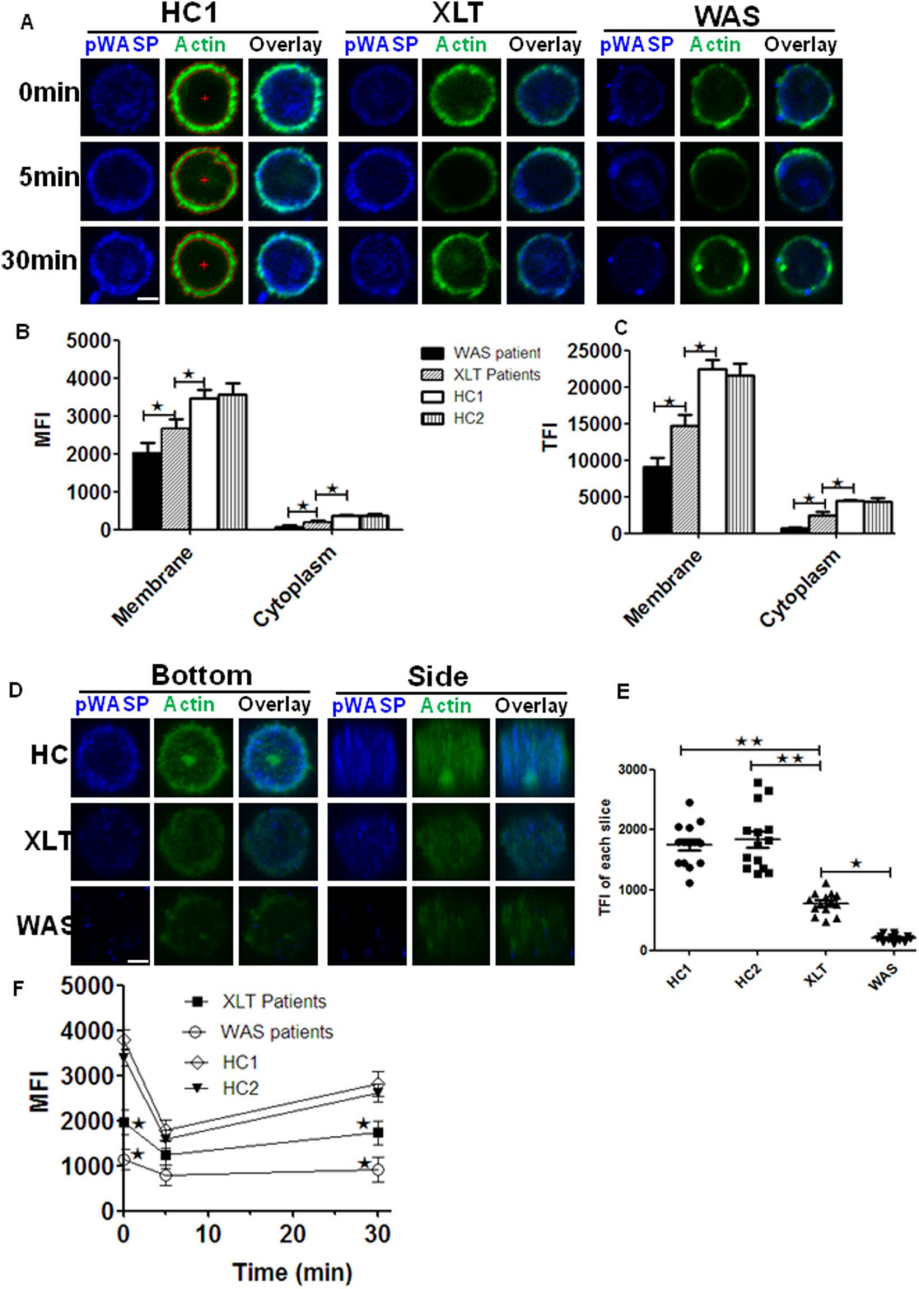
Actin organization is altered in XLT and WAS patients. (**A**) Sorted CD4^+^ T cells from HC, XLT and WAS patients were stained with phalloidin and antibodies specific for pWASP after stimulation with PMA and Ionomycin. Representative images from WAS patients and XLT patients are shown. (**B** and **C**) The quantification of MFI or TFI of actin on the membrane and in the cytoplasm from 100 T cells evenly of 5 HC1s, 5 HC2s, 4 XLT and 4 WAS patients. (**D**) 3D images of T cells stained with pWASP and phalloidin without stimulation from bottom and side view. (**E**) Quantification of the TFI of actin in each slice of T cells from a representative of HCs, XLT (P5) and WAS patients (P1). (**F**) Flow cytometry analysis of the MFI of F-actin of T cells upon stimulation with PMA and Ionomycin from 5 HC1s, 5 HC2s, 4 XLT and 4 WAS patients. Scale bar, 0.25 um. *P < 0.01.

WASp is a regulator of actin cytoskeleton, which is tightly related with cell migration. Therefore we examined the actin organization in WAS and XLT patients by using immunofluorescence microscopy and flow cytometry upon stimulation with PMA and Ionomycin. We found the MFI or TFI of F-actin determined by NIS-Elements AR 3.2 software on the membrane and in the cytoplasma was reduced in XLT patients and more reduced in WAS patients when compared to control group HC1 or HC2 (Fig. 4A–C). We took the 3D images of the T cells from HC1, HC2, XLT and WAS patients and quantified the F-actin level of each slice with NIS-Elements AR 3.2 software. We found the F-actin level of each slice in HC1 or HC2 group was significantly higher than that of XLT group, and that of XLT group was also significantly higher than that of WAS group (Fig. 4D and E). We further examined the mean fluorescence intensity (MFI) of actin during T - cell activation by using flow cytometry. The MFI of actin decreased at 5 min for all the samples, which indicates the actin depolymerization. The MFI of actin increased afterwards until 30 min, which indicates the actin polymerization. At 30 min, we found the levels of F-actin in XLT were decreased and further decreased in WAS compared to that of control group HC1 or HC2 (Fig. 4F). All these results collectively imply that the degree of actin alteration is inversely associated with WASp expression and indicate the disrupted actin organization may account for thymic output.

## Discussion

In this study, we diagnosed eight patients, four classic WAS and four XLT, with their clinical manifestations and *WAS* gene mutations. We found that WASp expression were absent or decreased in WAS patients and decreased or normal in XLT paitients. We analyzed the percentages and numbers of T - cell subsets in the peripheral blood of eight patients and HCs, and found CD8^+^ T naïve cells of the classic WAS patients were significantly reduced. T cell lymphopenia in patients with WAS has been described before^10^. Decreased proliferation and increased apoptosis of WAS T cells has been considered the main reason for T cell lymphopenia^10,11^. Here we examined if altered thymic output may contribute to T cell lymphopenia in WAS. Although the thymus is an essential lymphoid organ for the output of naïve T cells, thymic output has not been measured adequately in WAS^12^. To determine the level of thymic output in WAS patients, we examined the sjTRECs of patients and age-matched HCs. We found decrease of thymic output in patients with classic WAS and XLT compared with HCs. Jikina *et al*. previously examined the TRECs copy numbers in one WAS and one XLT patient and found normal TRECs in the WAS and XLT patient when compared to a healthy donor^13^. TRECs assay is tightly related with the age and background of the individuals^14^. Jilkina *et al*. included only one WAS and XLT and it is difficult to make conclusions with limited samples. Our analysis now include four WAS and four XLT patients and shows reduced number of TRECs in patients. Due to the variability of each individual such as the genetic background and living environment, it is hard to make accurate conclusions sometimes. The TRECs analysis in mice is helpful to guarantee the results from patient samples. In addition to WAS patients, we also detected reduced TRECs in blood T cells from WAS KO mice and found similar results. However, the TRECs copy numbers are not that different between the two HC groups – this somehow does not correlate to the dramatic differences in T cell numbers. As known, the decrease of TRECs copy numbers is correlated with the reduction of T cell numbers in the periphery, but not the only reason for it. Other factors may contribute such as increased apoptosis.

We examined the mean fluorescence intensity (MFI) and the subcellular location of F-actin in T cells by immunofluorescence microscopy and flow cytometry with or without stimulation. We found the levels of F-actin in T cells of the patients were decreased on the plasma membrane and in the cytoplasm without stimulation. Although the actin goes through depolymerization and polymerization in both WAS and XLT patients like that of HCs, the levels of F-actin in WAS and XLT patients fail to reach that of HCs after stimulation. Previous research shown that thymic output is highly correlated with actin cytoskeleton and a mutation in CORO1A (S401fs) was associated with increased F-actin accumulation in T cells and severely defective thymic output^15^. These results suggest that WASp plays a critical role in thymic output, which may be correlated with the disrupted subcellular location of actin in both steady and activation state.

The precise mechanism of how WASp regulates subcellular location of F-actin in T cells and how actin cytoskeleton affects thymic output has been conducted using the WASp-deficient mice. Earlier studies have shown defects in cell migration in WAS patients, suggesting that there could be caused by a general defect of signaling pathways involving the regulation of T cell migration and thymic output. Recently the WASP binding protein (WIP) has been studied in B cells and the absence of WIP lesds to the impairment of CD19 activation following PI3K signaling, which is due to a distortion in the actin and tetraspanin networks that causes altered CD19 cell surface dynamics^16^. In T cells, a fraction of total synaptic F-actin selectively generated by WASp in the form of distinct F-actin ‘foci’ was identified. These foci are polymerized de novo by the T - cell receptor (TCR) proximal tyrosine kinase cascade, and facilitate PLCγ1 activation and subsequent cytoplasmic calcium ion elevation^17^. The combination of two-photon microscopy and techniques of cell tracking to directly observe T cell migration in the thymus of WAS KO mice should reveal how migration in thymus leads to altered thymic output in WAS KO mice. It would be interesting to observe T cell migration in the thymus of WAS KO and wildtype bone marrow chimeric mice to investigate whether defects of T cell migration in the thymus of WAS KO mice is T cells intrinsic.

Cell migration is a complicated process that requires orchestrating and coordination of many different molecules in time and space. The actin cytoskeleton plays a major role in this process. Cdc42, Rac and Rho are the Rho subfamily of Ras-like GTPases, which can induce filopodia, lamellipodia and stress fibres in cells. Cdc42 can regulate the actin cytoskeleton through activation of WASp^5^. WASp is a critical regulator of actin nucleation, and participates in the transduction of signals from the cell surface to the actin cytoskeleton, through the Arp2/3 complex^18^. The VCA domain of WASp is the functional unit that interacts with and activates the Arp2/3 complex to initiate filamentous actin (F-actin) polymerization. The Arp2/3 complex localizes at the leading edge of migrating cells where it nucleates branched actin filaments. In lamellipodia, the WASp-Arp2/3 pathways generate a branched filament array^6,19^. Therefore it would be of interest to study the abnormal behavior of other actin regulators such as Cdc42, Rac and Rho in WAS or XLT patients and to see if the disruption of these actin regulators is coincident with the disrupted thymic output in WAS or XLT patients.

Overall, our study suggests that WASp deficiency may contribute to decreased thymic output in patients with WAS, through the regulation of subcellular location of F-actin in the T cells.

## Materials and Methods

All experiments involving human and mice samples were approved by the Ethics Committee of the Children’s Hospital of Chongqing Medical University, and were performed according to the tenets of the Declaration of Helsinki and Principles of Laboratory Animal Care. All experiments in this study were performed using protocols approved by the authors’ institutional usage committee and following institutional and NIH guidelines and regulations.

### Patients and samples

Blood samples were collected from four WAS patients (P1-4), four XLT patients (P5-8) and age-matched healthy controls. Informed consents, including both study participation and publication of identifying information, were obtained from all the children’s parents.

### Nucleic acid isolation

Genomic DNA was extracted from whole blood using the QIAamp DNA Mini Kit (Qiagen GmbH, Hilden, Germany) according to the manufacturers’ instructions.

### PCR reaction and analysis of WAS gene mutations

Genomic DNA samples were amplified with primer pairs designed to span each exon and exon/intron junctions in accordance with the reaction condition as previously described^3^. PCR products were sequenced using the automated ABI PRISM 3100 Genetic sequencer (PE Applied Biosystems, Foster City, CA, USA). Mutations were ensured by sequencing in the forward and opposite direction.

### Detection of WASp expression

As previouslty described^20^, intracellular WASp expression in Peripheral blood mononuclear cells (PBMCs) was detected by flow cytometry analysis. After fixation and perforation, 2 × 10^6^ PBMCs in each tube were incubated with 0.25 mg/ml purified mouse anti-human WASp mAb (BD Pharmingen) or isotype-matched control mouse IgG2a mAb (BD Pharmingen) at 4 °C for 30 min. Then all the cells were incubated with 1:50 diluted PE-conjugated Rat anti-mouse IgG2a (BioLegend) and reacted at 4 °C for 30 min. The PE- labeled cells was assessed by flow cytometry.

### Subsets of T cell by flow cytometry analysis

Human T cells were surface stained with anti-CD3 Percp-cy5.5, anti-CD4 FITC, anti-CD8 BV510, anti-CD27 APC, anti-CD45RA PE-Cy 7 (Biolegend). CD4^+^/CD8^+^ T cells were sorted as CD3^+^CD4^+^/CD3^+^CD8^+^ cells. Central memory T cells were stained with CD27^+^CD45RA^−^. Effector memory T cells were stained with CD27^−^CD45RA^−^. Naïve T cells were stained with CD27^+^CD45RA^+^. Terminally T cells were stained with CD27^−^CD45RA^+^.

### Quantification of signal joint TCR rearrangement excision circle (sjTRECs)

The quantitive measurement of sjTRECs in human CD3^+^ T cells was based on real-time PCR as reported^21^. The nuclear DNA samples and the probe (10^8^ copies/ml) undergo a first PCR amplification of 22 cycles using the out primers for both sjTRECs and CD3γ gene in the same reaction. Then the amplified products of probe were diluted by gradient dilution and undergo the second round of PCR to generate the standard curves. The amplified products of DNA samples were diluted by 100 times before conducting the second round of PCR using in primers. All the samples were run in triplicate. All analyzed qRT-PCR assays fulfilled the quality requirements of similar slopes and *R* ^2^ values of 0.99–1.0. Copy numbers of sjTRECs per 1 × 10^5^ CD3^+^T cells were presented.

Mice sjTRECs analysis in CD3^+^ T cells was performed as described^22^. We used real-time quantitative PCR (RQ-PCR) to detect sjTRECs in 8 weeks old WAS KO mice and WT mice peripheral blood leukocytes. PCR was performed in a 20 ul reaction, 95 °C for 10 min, the 95 °C for 15 secs, 60 °C for 1 min and 68 °C for 30 secs by 40 cycles. The PCR efficiencies were tested by a standard cueve. CD45^+^ and CD3^+^ subsets in peripheral blood were analyzed by flow cytometry.

### Immunofluorescence and flow cytometry

Sorted T cells from the PBMCs of the WAS patient (P1), the XLT patient (P5), and the Healthy control (HC) were incubated with 10 ng/ul Phorbol-12-myristate-13-acetate (PMA) plus 100 ng/ul ionomycin (sigma,Shanghai,China) at 37 °C for indicated times (0, 5, 30 min). Cells were fixed, permeabilized, and stained for pWASP and F-actin. The subcellular location and the mean fluorescence intensity (MFI) of F-actin in T cells were determined by confocal microscopy and flow cytometry. To do the quantification of the actin expression from the images of confocal microscopy, the membrane region of T cells was defined with NIS elements software based on the actin staining. The MFI of actin on the membrane and in the total cell was quantified by NIS elements software, and the MFI of actin in the cytoplasm was obtained from the MFI of actin in the total cells subtracted with the MFI of actin on the membrane.

## References

[1] Gulacsy V (2011). Genetic characteristics of eighty-seven patients with the Wiskott-Aldrich syndrome. Molecular immunology.

[2] Aldrich RA, Steinberg AG, Campbell DC (1954). Pedigree demonstrating a sex-linked recessive condition characterized by draining ears, eczematoid dermatitis and bloody diarrhea. Pediatrics.

[3] Zhu Q (1995). The Wiskott-Aldrich syndrome and X-linked congenital thrombocytopenia are caused by mutations of the same gene. Blood.

[4] Notarangelo LD, Miao CH, Ochs HD (2008). Wiskott-Aldrich syndrome. Current opinion in hematology.

[5] Kim AS, Kakalis LT, Abdul-Manan N, Liu GA, Rosen MK (2000). Autoinhibition and activation mechanisms of the Wiskott-Aldrich syndrome protein. Nature.

[6] Le Clainche C, Carlier MF (2008). Regulation of actin assembly associated with protrusion and adhesion in cell migration. Physiological reviews.

[7] Trifari S (2006). Defective Th1 cytokine gene transcription in CD4+ and CD8+ T cells from Wiskott-Aldrich syndrome patients. Journal of immunology.

[8] Morales-Tirado V (2010). Critical requirement for the Wiskott-Aldrich syndrome protein in Th2 effector function. Blood.

[9] Sarzotti-Kelsoe M (2009). Thymic output, T-cell diversity, and T-cell function in long-term human SCID chimeras. Blood.

[10] Park JY (2004). Early deficit of lymphocytes in Wiskott-Aldrich syndrome: possible role of WASP in human lymphocyte maturation. Clinical and experimental immunology.

[11] Rawlings SL (1999). Spontaneous apoptosis in lymphocytes from patients with Wiskott-Aldrich syndrome: correlation of accelerated cell death and attenuated bcl-2 expression. Blood.

[12] Ribeiro RM, Perelson AS (2007). Determining thymic output quantitatively: using models to interpret experimental T-cell receptor excision circle (TREC) data. Immunological reviews.

[13] Jilkina O (2014). Retrospective TREC testing of newborns with Severe Combined Immunodeficiency and other primary immunodeficiency diseases. Molecular genetics and metabolism reports.

[14] Zhang SL, Bhandoola A (2012). Losing TREC with age. Immunity.

[15] Yee CS (2016). Recurrent viral infections associated with a homozygous CORO1A mutation that disrupts oligomerization and cytoskeletal association. The Journal of allergy and clinical immunology.

[16] Keppler SJ (2015). Wiskott-Aldrich Syndrome Interacting Protein Deficiency Uncovers the Role of the Co-receptor CD19 as a Generic Hub for PI3 Kinase Signaling in B Cells. Immunity.

[17] Kumari, S. *et al*. Actin foci facilitate activation of the phospholipase C-gamma in primary T lymphocytes via the WASP pathway. *eLife* **4**, doi:10.7554/eLife.04953 (2015). 10.7554/eLife.04953 PMC4355629 25758716

[18] Massaad MJ, Ramesh N, Geha RS (2013). Wiskott-Aldrich syndrome: a comprehensive review. Annals of the New York Academy of Sciences.

[19] Goley ED, Welch MD (2006). The ARP2/3 complex: an actin nucleator comes of age. Nature reviews. Molecular cell biology.

[20] Liu DW (2015). Wiskott-Aldrich syndrome/X-linked thrombocytopenia in China: Clinical characteristic and genotype-phenotype correlation. Pediatric blood & cancer.

[21] Dion ML, Sekaly RP, Cheynier R (2007). Estimating thymic function through quantification of T-cell receptor excision circles. Methods in molecular biology.

[22] Broers AE (2002). Quantification of newly developed T cells in mice by real-time quantitative PCR of T-cell receptor rearrangement excision circles. Experimental hematology.

